# An analysis of the UK national pancreas allocation scheme

**DOI:** 10.3389/frtra.2024.1408838

**Published:** 2024-08-15

**Authors:** Jeevan Prakash Gopal, Sean P. Gavan, Kerry Burke, Stephen Birch, Titus Augustine

**Affiliations:** ^1^ Manchester Centre for Transplantation, Manchester Royal Infirmary, Manchester University NHS Foundation Trust, Manchester, United Kingdom; ^2^ Manchester Centre for Health Economics, Division of Population Health, Health Services Research and Primary Care, School of Health Sciences, Faculty of Biology, Medicine and Health, The University of Manchester, Manchester, United Kingdom; ^3^ Department of Vascular Surgery, Manchester Royal Infirmary, Manchester University NHS Foundation Trust, Manchester, United Kingdom; ^4^ Centre for Business and Economics of Health, University of Queensland, Brisbane, QLD, Australia; ^5^ Faculty of Biology, Medicine and Health, Division of Diabetes, Endocrinology and Gastroenterology, University of Manchester, Manchester, United Kingdom

**Keywords:** beta cell replacement, pancreas transplantation, islet transplantation, pancreas allocation scheme, organ utilisation

## 1 Introduction

Modern technology-driven health care delivery strategies require robust health economic analyses in order to ensure best use is being made of available resources. This is of particular importance in a system like the National Health Service (NHS). Organ transplantation in the NHS, is delivered through NHS Blood and Transplant (NHSBT) using organ allocation policies which have evolved with clinical advances, and which are revised periodically. The fundamental principles which drive the different organ allocation policies are equity of access to good quality organs while achieving optimal recipient outcomes with the least waiting times. The transplant pathway is particularly complex, with the main constrained resource being the availability of suitable donor organs. While clinical priorities and outcomes are paramount in organ allocation policies for transplantation, economic estimates also play a vital role by providing an indication of the price or cost considerations that can help inform decision makers about optimal resource utilisation especially when faced with limited organ availability and competing organ demands. This is particularly evident in beta cell replacement for Type 1 Diabetes Mellitus where two transplant options exist: solid organ pancreas transplantation and allogenic islet cell transplantation both of which are delivered by NHSBT as clinical programmes.

Diabetes mellitus is a health epidemic that is unique, by the sheer breadth and impact of its consequences globally, nationally, individually and economically (1). The global economic burden due to diabetes is projected to increase from U.S. $ 1.3 trillion in 2015 to U.S. $ 2.1 trillion by 2030 (1). While insulin continues to be the mainstay of diabetes therapy, technological advancements in insulin delivery have significantly improved the management of all types of diabetes. This is particularly evident in Type 1 diabetes where insulin delivery mechanisms have evolved exponentially into highly complex and effective glucose sensing and insulin pump therapy techniques (2, 3).

Beta cell replacement therapy (solid organ pancreas and islet cell transplantation) however is the therapeutic technique closest to mimicking the body's natural closed loop mechanism of glucose homeostasis. Globally there are significant numbers of patients with diabetes and end stage kidney failure due to diabetic nephropathy. Simultaneous pancreas and kidney transplantation is considered the gold standard treatment for patients with diabetes and end stage renal failure, albeit at the expense of major surgery and lifelong immunosuppression (4–6). Pancreas transplantation alone is also an effective intervention for severe Type 1 diabetic patients, suitable for and willing to undergo a major surgical procedure for severe incapacitating diabetes along with lifelong immunosuppression.

Islet transplantation is another technique that is minimally invasive, of lesser magnitude and with lower procedure related risks than whole pancreas transplantation. Since the first successful trial of human islet cell transplants in 1990 (7), this beta cell replacement technique has developed at a slower pace than solid organ pancreas transplantation due to the technical difficulties associated with islet isolation and obtaining an adequate islet mass for transplantation. Islet transplantation is aimed to benefit a specific cohort of patients who are different to those listed for solid organ pancreas transplantation, and is not really a competing procedure (8) as the therapeutic end point is primarily focused on managing difficult hypoglycaemia unawareness.

Although islet transplantation was first pioneered in North America, clinical islet transplantation is still considered as an experimental procedure in the United States due to the regulatory barriers, and there has been a decline in the number of islet transplants performed in the recent years (9, 10). In the United Kingdom (UK), islet transplantation is a recognised clinical therapeutic intervention, by the National Institute for Health and Care Excellence (NICE) (11) and is delivered as a highly specialised service, commissioned nationally and fully reimbursed by the NHS through established reimbursement mechanisms with the designated transplant centres.

The aim of this analysis was to evaluate the performance of the current allocation policy according to the relative number of whole organ or islet transplants that can be performed from the limited supply of donor pancreases. Retrospective data for all pancreases retrieved for transplantation and subsequently allocated to either transplant technique was analysed to estimate the rate of transformation (or trade-off) of retrieved organs to transplants between the two techniques. Initial headline data between 01/04/08 and 31/03/2016 were provided by NHSBT on request. Two formal requests for detailed and complete data were refused. Hence data for April 2016 until March 2022 were obtained from nationally available reports.

## 2 Assessment of policy and its implications

National commissioning of pancreas transplantation in the United Kingdom was first implemented in Scotland in 2000. The UK pancreas transplantation taskforce was established in 2002 to develop pancreas transplantation in the UK leading to national commissioning of the service in England in 2004 and Wales in 2006. A clinical islet cell transplantation programme was first commissioned as a national service in England in 2008, followed by Scotland in 2009. The UK Islet Transplant Consortium (UKITC) has established a collaborative “hub and spoke” model with 3 islet isolation centres and 7 islet transplant centres (12). Both solid organ pancreas and islet transplantation are delivered by 4 centres. There are only two 2 centres which isolate and transplant both solid organ and islets.

### 2.1 The UK national pancreas allocation scheme

In 2002 the pancreas transplantation taskforce allocated pancreases through a scheme which had several shortcomings due to zonal allocation. The national pancreas allocation scheme (13), developed by the pancreas transplantation taskforce, replaced it in December of 2010. The UK national pancreas allocation policy (13) allocates donor pancreases to recipients in both the solid organ and the islet transplant waiting lists by a principle of equal access.

The national pancreas allocation scheme awards points to patients for the following factors: total HLA (Human Leukocyte Antigen) mismatch, recipient waiting time, recipient sensitisation status, organ travel time, donor body mass index (BMI), recipient dialysis status, and donor to recipient age matching. Pancreas from Donation after Brainstem Death donors <61 years can be allocated for islet or solid organ transplants, whereas for Donation after Circulatory Death pancreas, the donor age cut off for allocation to solid organ transplant is <56 years and islet transplant is <51 years. Pertaining to the donor BMI and age, pancreas from donors with BMI of <25 and age <25 years will not be initially offered for islet transplantation, and pancreas from donor with BMI of >31 will not be initially offered for solid organ transplantation. Pancreas from donors with BMI between 26 and 30 can be allocated for solid organ or islet transplant. The allocation algorithm then calculates the total points score and the patient with the highest total point score will be ranked first in the offering sequence (13).

This national allocation scheme is unique for the following reasons. Firstly, it is a recipient-oriented equitable model for organ allocation as recipients on the islet transplant waiting list have equal access to donor organs of the same quality as recipients on the solid organ pancreas waiting list. Secondly, it is a predominantly data driven scheme that was developed based on the UK and the United States registry datasets on outcomes and waiting lists. Finally, it has been instrumental in bringing pancreas and islet transplantation together as complementary treatment options under the banner of beta-cell replacement rather than two competing treatment modalities.

Analysis of the scheme after 3 years since its introduction showed that the results were approximately as simulated, with a significant reduction in the number of long waiting patients and an increase in the islet transplant activity without impacting the solid organ pancreas transplant activity (14).

While clinical outcomes after pancreas and islet transplantation are regularly analysed and published in annual national reports, the economic implications of the allocation policy in the context of pancreas utilisation has not been investigated. This is pertinent because there are two different procedures that are competing for the same scare resource (donor pancreases) with differing treatment goals. A further important context is the magnitude of diabetes as a national problem, and the limited number of individuals for whom the pancreas or islets can be effectively transplanted. A full economic evaluation comparing the resource requirements, costs, and health benefits would provide additional information on effective utilisation which could be used for future allocation modelling or modifying the existing allocation policy.

### 2.2 Pancreas and islet transplantation: activity and outcomes in the UK

A total of 2,779 solid organ pancreas transplants (406 pancreas only transplants) have been performed from 1st April 2007 to 31st March 2022 across eight centres and 336 islet cell transplants (including 29 simultaneous islet kidney transplants) have been performed from 1st April 2008 to 31st March 2022 across seven centres (15).

Outcomes are shown in Table 1. While there is still debate of the definition of graft failure, currently failure is defined as return to permanent insulin dependence for solid organ pancreas transplant and C-peptide <50 pmol/L for islet graft failure.

**Table 1:** Outcomes of pancreas and islet transplantation^b^.

Outcome/Parameter	Period	Pancreas transplantation	Islet transplantation
Median active patient waiting time	2016–2020	359 days	336 days (Routine graft)
1-year graft survival	2017–2021	87% (CI: 68–95) for PTA (Pancreas Transplant Alone)	76% (CI: 55–88) for Routine graft
5-year graft survival	2013–2017 (PTA) & 2012–2021 (Islet transplant)	66% (CI: 52–76) for PTA	38% (CI: 22–54) for routine only graft 67% (CI: 54–78) for routine followed by priority graft 57% (CI: 33–75) for routine only graft^a^ 73% (CI: 58–83) for routine followed by priority graft^a^
1-year patient survival	2013–2017	100% for PTA	Not reported
5-year patient survival	2013–2017	88% (71–95) for PTA	Not reported
NHS England's total expenditure for providing service	2019–2020	£5–10 m (for estimated 131 transplants)	£1–5 m (for estimated 15 transplants)

^a^ Routine graft was functioning at 1-year.

^b^ Reference (15).

For routine only, islet transplants performed between 2017 and 2021, the median annual rate of severe hypoglycaemic events was 14 events pre-transplant, compared to 0 events at 1-year post-transplant. The median HbA1c (mmol/mol) dropped from 68 mmol/mol pre-transplant to 56 mmol/mol at 1-year post-transplant. There was also a reduction in the median insulin dose/kg recipient body weight at three-months (0.25 Units/kg/day), six-months (0.24 Units/kg/day) and 1-year post-transplant (0.28 Units/kg/day) compared to pre-transplant (0.43 Units/kg/day). Insulin independence was achieved in 53% of the recipients at some point during the 1st year post-transplant (16).

### 2.3 Organ utilisation

**Figure 1:**
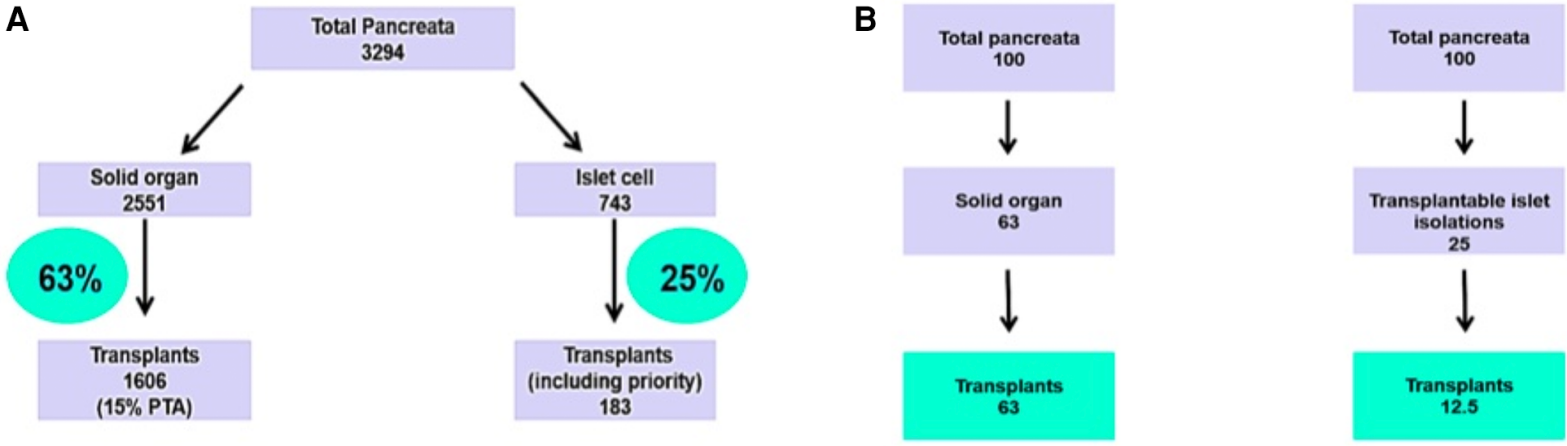
(**A**) Pancreata allocation between 01/04/08 and 31/03/2016. NHS Blood and Transplant national data. (**B**) Analytical framework of allocation of a fixed supply of pancreases shared between solid pancreas and islet transplants, according to utilisation data.

Between 1st April 2008 and 31st March 2016, 3294 pancreases were retrieved for solid organ and islet cell transplantation. Of these, 2551 entered the solid organ pathway, with 1,606 transplants being performed (63% utilisation) the remaining 743 entered the islet pathway, with 183 transplants (25% utilisation) (15). The standard practice is that islet transplant recipients should receive a second islet transplant if needed within 3 months of the first transplant (priority graft) (Figure 1A).

Figure 1B illustrates the number of patients who would be treated based on these utilisation rates in a scenario where there is a supply of 100 pancreata available. If all 100 pancreata are allocated to solid organ transplantation, then 63 patients would receive a transplant. Alternatively, if all 100 pancreata are allocated to islet cell transplantation, then 12 patients would receive a transplant (assuming that all islet patients require two islet transplants). For every recipient of an islet cell transplant, the same organs could have been used on average for five solid pancreas transplant recipients.

Between 1st April 2016 and 31st March 2022, a total of 2,310 pancreases were retrieved for solid organ and islet transplantation, which led to 932 solid organ transplants (79 pancreas only transplants) and 154 islet transplants (including 29 simultaneous islet kidney transplants). Information pertaining to organ allocation to solid organ or islet transplant was not published in the annual reports (16).

### 2.4 Actionable recommendation

While this analysis focuses on wastage in the islet arm, wastage also occurs in the solid organ pancreas transplant group due to anatomical and other issues. These are grafts from which islets could potentially be isolated. An important islet source are good solid pancreases not utilised due to anatomical reasons. It may be that re-organisation of the national services into unified combined centers (solid organ pancreas and islet transplant centers/islet isolation facilities) could lead to less wastage in both techniques.

## 3 Discussion

Diabetes Mellitus is a global health problem with far reaching economic consequences. The international diabetes federation has predicted the prevalence of diabetes to increase from 415 million in 2015 to 783 million by 2045 (17). In the UK, diabetes affects one in sixteen people with 700 individuals newly diagnosed daily. The number of pancreas and islet transplants performed forms a tiny fraction of patients with diabetes nationally and potentially eligible for beta cell replacement. It is estimated that £10 billion (10% of the NHS financial budget) is spent annually on the direct cost of diabetes, of which £1 billion is spent on type 1 diabetes. If indirect costs are also considered, it is £23.7 billion (18–20).

Pancreas and islet transplantation are fully reimbursed nationally commissioned services delivered through designated centres. All nationally commissioned services have been robustly costed to establish representative tariffs. NHS England's total expenditure during 2019/20 for providing Islet transplantation service was between £1million and £5 million for an estimated number of 15 transplants whereas for whole organ pancreas transplantation the expenditure was between £5 and £10 million for an estimated 131 transplants (21).

Islet cell transplantation is primarily intended for diabetic patients with disabling hypoglycaemia unawareness which has not responded to maximal medical therapy. It has the potential to reduce the number of hypoglycaemic episodes, improve glucose control, reduce insulin requirements and indirectly improve patient's quality of life. The single largest advantage of islet transplantation, is the lower magnitude of procedure related morbidity and mortality when compared to solid pancreas transplantation, which requires invasive major surgery associated with much higher morbidity and mortality. However solid organ pancreas transplants if successful provide insulin independence. While the donor pancreas is the starting point for both procedures, the two differing end points are starkly represented by the definition of graft failure.

Islet transplantation outcomes have improved, with recent studies publishing long term data (22). As the joint organ allocation policy provides access to better quality donor pancreas for islet transplantation, outcomes in the recent years in the UK have improved. Although, there is a marked reduction in the incidence of hypoglycaemia unawareness and improved metabolic control, the medium- or long-term insulin independence rate achieved by islet transplantation is not comparable to solid organ pancreas transplantation. It is recognised by the beta cell replacement community that achieving insulin independence is not the primary aim of islet transplantation.

To achieve this end point however, usually more than one transplant is required, for each recipient. This has led to the search for alternative sources for islet cells for transplantation (23, 24). From a health economic perspective, it is important to critically appraise the cost and health benefits of using a greater amount of quality donor pancreases for the relatively limited outcome from islet cell transplantation compared to solid organ pancreas transplantation.

The pancreas remains one of the least utilised organs among solid organ transplantation. UK donation and transplantation rates of organs from donation after brainstem death (DBD) donors between 1st April 2021 and 31st March 2022, show that only 17% of pancreases from DBD donors were transplanted. This is similar to lung, 17% and higher than bowel (3%) and heart 10% (25).

Utilisation concerns in transplantation are universal and in the United Kingdom the Department of Health and Social Care have established a utilisation group to review organ utilisation (26). It is universally evident that currently islet transplantation is unsustainable from the perspective of donor pancreas utilisation and islet isolation. The collaborative islet transplant registry published their data report compiling data from 1999 to 2020 with cumulative North American, Australian and Eurasian data (22). A total of 1,399 allogenic islet transplants were performed using 2,832 infusions from 3,326 donors (22). This approximates to the use of nearly 3 donor pancreases for 1 clinically successful islet transplant.

Deceased donor pancreases for transplantation are scarce and exceeded by the demand for donor pancreases leading to many challenges. While surgical techniques and technological therapeutic advances have continued to evolve exponentially, the benefits of this depends on translation to equitable access, whereby treatments should be available of equal quality to all people with needs for treatment without respect to gender, ethnicity, geographical location or socioeconomic status. Although the joint allocation policy provides equitable access to pancreas and islet transplant waiting list patients, the use of donated organs may not be efficient with respect to maximising health outcomes from the limited organs available.

The concept of wide-spread delivery of a scarce resource by the national pancreas allocation policy is attractive both in practice and in principle. NHSBT's allocation policies for many organs regularly provide preferential weight for particular groups, such as children, uncommon blood groups (such as in ethnic minorities) and highly sensitised persons, all with the purpose of maximising overall community access. Islet transplantation, where clinically indicated, can be seen as having the same purpose, particularly since the numbers actually involved are low. Relinquishing an equal access policy would have the effect, as in the United States, of decreasing the number of islet cell transplants, thus providing less opportunity for delivery and development of the technique. It is the overall cost-effectiveness and resource utilisation of islet cell transplantation that needs to be revisited.

The allocation system needs to ensure efficient use of limited organ supplies and at the same time allow equitable access for different patient groups. Undoubtedly islet cell transplantation has an important role in beta cell replacement therapy. Although inferior in achieving insulin independence and costs to solid organ transplantation, it does confer significant benefits in a specific subset of patients. However, for a less comprehensive end-point, islet cell transplantation is an expensive treatment option, and potentially utilises more than one pancreas per recipient. This adds to the burden of organ scarcity which in turn is compounded by organ wastage in the solid pancreas transplant category.

The main limitation of the study is lack of comprehensive data (to perform a health economic analysis) from April 2016 to March 2022, especially the number of organs that went into each treatment arm and the utilisation rates. Two formal requests for data for a full health economic analysis was declined by the Pancreas Advisory Group.

The national pancreas allocation policy is unique in its design and delivers its strategic ambition of two separate interventions from one resource, a donor pancreas. As currently delivered, it brings into focus the complexities of managing extremely scarce donor organs with the best outcomes for a common disease, while trying to adhere to the main commissioning principles of a nationally commissioned service, which require fairness and consistency throughout the country, ensuring that patients have equitable access to the services regardless of their location. However, after more than a decade of service delivery, islet transplant continues to be an expensive procedure benefitting a small number of patients with a disproportionate requirement of donor pancreases and potential organ wastage in the islet arm. While this analysis does not consider the ethical perspective of donor organ wastage, from a cost-effectiveness and effective resource utilisation perspective, it may be time for a review and radical reorganization.
